# Manganese-contaminated groundwater treatment by novel bacterial isolates: kinetic study and mechanism analysis using synchrotron-based techniques

**DOI:** 10.1038/s41598-020-70355-w

**Published:** 2020-08-07

**Authors:** Nakharin Therdkiattikul, Thunyalux Ratpukdi, Pinit Kidkhunthod, Narong Chanlek, Sumana Siripattanakul-Ratpukdi

**Affiliations:** 1 Department of Environmental Engineering, Faculty of Engineering and Research Center for Environmental and Hazardous Substance Management, Khon Kaen University, Khon Kaen, 40002 Thailand; 2 Center of Excellence On Hazardous Substance Management (HSM), Bangkok, 10330 Thailand; 3 Synchrotron Light Research Institute (Public Organization), Nakhon Ratchasima, 30000 Thailand

**Keywords:** Pollution remediation, Water microbiology, Characterization and analytical techniques

## Abstract

The occurrence of manganese in groundwater causes coloured water and pipe rusting in water treatment systems. Consumption of manganese-contaminated water promotes neurotoxicity in humans and animals. Manganese-oxidizing bacteria were isolated from contaminated areas in Thailand for removing manganese from water. The selected bacterium was investigated for its removal kinetics and mechanism using synchrotron-based techniques. Among 21 isolates, *Streptomyces violarus* strain SBP1 (SBP1) was the best manganese-oxidizing bacterium. At a manganese concentration of 1 mg L^−1^, SBP1 achieved up to 46% removal. The isolate also successfully removed other metal and metalloid, such as iron (81%) and arsenic (38%). The manganese concentration played a role in manganese removal and bacterial growth. The observed self-substrate inhibition best fit with the Aiba model. Kinetic parameters estimated from the model, including a specific growth rate, half-velocity constant, and inhibitory constant, were 0.095 h^−1^, 0.453 mg L^−1^, and 37.975 mg L^−1^, respectively. The synchrotron-based techniques indicated that SBP1 removed manganese via combination of bio-oxidation (80%) and adsorption (20%). The study is the first report on biological manganese removal mechanism using synchrotron-based techniques. SBP1 effectively removed manganese under board range of manganese concentrations. This result showed the potential use of the isolate for treating manganese-contaminated water.

## Introduction

Manganese is an abundant transition metal that disperses readily through soil and water. It can exist in many oxidation states (from Mn^3−^ to Mn^7+^). In aquatic environments, the main forms are dissolved (Mn^2+^) and oxidized (Mn^3+^ and Mn^4+^) manganese. The presence of manganese in groundwater is a common problem in many countries^1^. In the USA, high manganese concentrations (up to 5.6 mg L^−1^) have been reported in numerous groundwater wells (68% of monitored wells)^2^. In Vietnam and China, manganese concentrations of approximately 1.2 mg L^−1^ were found in groundwater^3, 4^. The mentioned concentrations were much higher than the allowable concentrations for drinking water and water supplies in those countries. The United States Environmental Protection Agency sets the manganese standard at 0.05 mg L^−1^ for drinking water, while the World Health Organization allows a manganese concentration of 0.1 mg L^−1^ in a water supply^2, 5^. In a water treatment system, the presence of manganese (0.1 mg L^−1^ or greater) can cause aesthetic problems and pipe rusting^6^. In addition, a high manganese concentration (0.2 mg L^−1^ or greater) can cause neurotoxicity in humans and animals including Parkinson’s symptoms, emotional instability, and hallucinations^7–10^. Traditional manganese removal is performed in water treatment facilities by transforming soluble manganese to an insoluble form through oxidation, then separating the insoluble manganese via sedimentation and/or filtration processes. However, in many cases, chemical oxidation processes have not been adequate for removing manganese to drinking water standards.

Biological oxidation by microorganisms has been considered as an alternative method for manganese removal from water^11^. The advantages of biological manganese removal for water treatment include high manganese oxidation performance, easy installation in the treatment system, low cost, and minimal or no chemical utilization^12^. To accelerate the biological process, contaminant-removing microorganisms are isolated and augmented in the contaminated environment. Manganese-oxidizing bacteria have been isolated from soil, water pipes, and sediment in groundwater wells in many countries. Previously studied bacteria include strains in the genera *Bacillus*, *Lepthothrix*, *Pseudomonas*, *Roseobacter*, and *Acinetobacter*^13–17^. Among the isolates, *Leptothrix* spp., *Bacillus* spp., and *Acinetobacter* sp. strain LB1 have been applied to the removal of manganese from contaminated water^17–19^. The manganese oxidation performance reported in previous works has varied based on bacterial growth, manganese removal capability, and the tested environmental conditions. In addition, the manganese removal mechanism to support the manganese removal system, whether adsorption or oxidation by bacterial cells, was proposed based on indirect measurement^20–22^.

To treat manganese contamination in water, manganese-oxidizing bacteria could be inoculated to accelerate the treatment efficiency. In practice, the previously isolated cultures may not survive well or perform effectively in a different environment. Therefore, using an indigenous culture isolated from the contaminated site could be more promising for manganese oxidation for water treatment. The isolated culture should be investigated for its removal performance and mechanism to better understand its potential and limitations. Thus far, there has been no published work reporting bacterial isolation and its removal kinetics for manganese treatment.

This work aimed to isolate manganese-oxidizing bacteria from a manganese-contaminated area in Thailand. The bacterial species were identified and their manganese removal performances assessed. Monod and self-substrate inhibition kinetic models were constructed for the manganese-oxidizing bacterium selected based on performance. Removal of manganese and other metal and metalloid (iron and arsenic) by the novel isolated culture was demonstrated. Also, manganese removal from real groundwater was demonstrated. The micro-structure and potential manganese removal mechanism were characterised using microscopic and advanced spectroscopic techniques, including scanning electron microscopy coupled with energy-dispersive x-ray spectroscopy (SEM–EDS), X-ray photoelectron spectroscopy (XPS), and X-ray absorption spectroscopy (XAS)^23–25^. To the best of our knowledge, the current investigation represents the first report on the fundamental manganese removal mechanism of an isolated bacterium using synchrotron-based techniques.The bacterial isolate could be used for water treatment. Also, the fundamental information obtained through this work is useful for further applications.

## Results

### Manganese-oxidizing bacterial enrichment and isolation

Eight and nine bacterial colonies, respectively, were enriched from the soil (named SBP) and groundwater filter medium (designated FBP) samples taken from Ban Phai district (Khon Kaen, Thailand). Another soil source (named SKN) from Kranuan district (Khon Kaen, Thailand) yielded four isolates. The colony morphology of the 21 isolates is shown in Table 1. Based on the formulation of the bacterial medium, with a manganese concentration of 100 mg L^−1^, the isolates were manganese-tolerant bacteria and had potential for manganese oxidation.

**Table 1:** LBB spot test and colony morphology of enriched bacterial cells.

Name	LBB spot test					Size (mm)	Colony morphology at 48 h				Environmental medium	Location
	1	2	3	4	5		Form	Colour	Elevation	Margin		
**SBP1**	**+**	**+**	**+**	**+**	**+**	**1**	**Circular**	**White**	**Umbonate**	**Entire**	**Soil**	**Ban Phai**
**SBP2**	**+**	**+**	**+**	**+**	**+**	**1**	**Circular**	**White**	**Umbonate**	**Entire**	**Soil**	**Ban Phai**
**SBP3**	**+**	**+**	**+**	**+**	**+**	**1**	**Circular**	**White**	**Umbonate**	**Entire**	**Soil**	**Ban Phai**
SBP4	−	−	−	+	−	1	Irregular	Yellow	Flat	Undulate	Soil	Ban Phai
SBP5	−	−	−	−	−	1	Circular	Yellow	Convex	Entire	Soil	Ban Phai
SBP6	−	−	−	−	−	1.5	Circular	Yellow	Convex	Entire	Soil	Ban Phai
**SBP7**	**+**	**+**	**+**	**+**	**+**	**1**	**Circular**	**Yellow**	**Convex**	**Entire**	**Soil**	**Ban Phai**
SBP8	−	−	−	−	−	0.3	Circular	Yellow	Convex	Entire	Soil	Ban Phai
FBP1	−	−	−	−	−	0.5	Circular	Orange	Convex	Entire	Sand filter	Ban Phai
FBP2	−	−	−	−	−	1.5	Circular	Yellow	Convex	Entire	Sand filter	Ban Phai
**FBP3**	**+**	**+**	**+**	**+**	**+**	**1**	**Circular**	**White**	**Convex**	**Entire**	**Sand filter**	**Ban Phai**
FBP4	−	−	+	−	−	1.5	Circular	Yellow	Convex	Entire	Sand filter	Ban Phai
FBP5	−	−	−	−	−	0.5	Circular	Yellow	Convex	Entire	Sand filter	Ban Phai
FBP6	−	−	−	−	−	1	Circular	Yellow	Convex	Entire	Sand filter	Ban Phai
FBP7	−	−	+	+	−	1	Circular	Brown	Pulvinate	Entire	Sand filter	Ban Phai
FBP8	−	−	−	−	−	0.5	Irregular	Yellow	Flat	Undulate	Sand filter	Ban Phai
FBP9	−	+	−	+	−	1	Circular	Brown	Pulvinate	Entire	Sand filter	Ban Phai
SKN1	−	−	−	−	+	0.5	Circular	Yellow	Convex	Entire	Soil	Kranuan
SKN2	−	−	−	−	−	0.5	Circular	White	Convex	Entire	Soil	Kranuan
**SKN3**	**+**	**+**	**+**	**+**	**+**	**1**	**Circular**	**White**	**Convex**	**Entire**	**Soil**	**Kranuan**
SKN4	−	−	−	−	−	0.5	Irregular	Yellow	Flat	Undulate	Soil	Kranuan

The manganese oxidation potential of the enriched cultures was investigated via the LBB method, as presented in Table 1, following Akob et al^26^. Manganese oxidation was positive (five replicates) for six (SBP1, SBP2, SBP3, SBP7, FBP3, and SKN3) of the twenty-one enriched cultures. These six isolates, enriched from different environmental media and sources, were promising for manganese oxidation and were further tested for manganese removal efficiency. During the 7-d experiment (at an initial manganese concentration of 5 mg L^−1^), the isolates removed between 5.72 and 28.08% of the initial manganese. Three of the six isolates, SBP1 (24.30 ± 2.05%), SBP3 (28.08 ± 1.98%), and SBP7 (10.48 ± 2.93%) (average ± standard deviation), exhibited the highest performances and were chosen for later experimentation.

### Manganese-oxidizing bacterial selection

**Figure 1:**
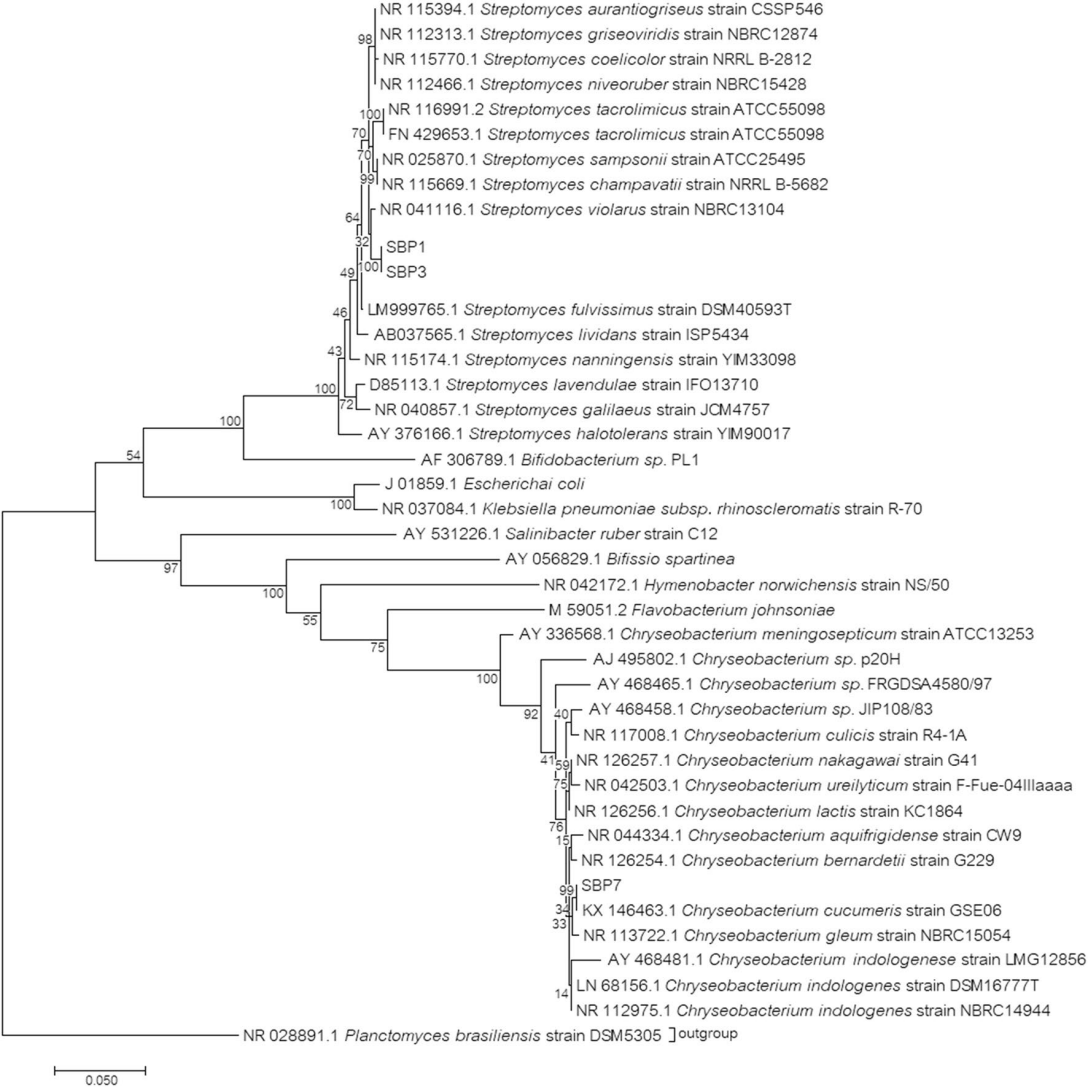
Neighbor-joining phylogenetic tree of 16S rRNA gene sequences.

Neighbour-joining phylogenetic analysis of the isolated cultures (SBP1, SBP3, and SBP7) was performed using the 16S rRNA gene nucleotide sequences (Fig. 1). SBP1, SBP3, and SBP7 had the highest similarity to *Streptomyces violarus* (98%), *Streptomyces violarus* (98%), and *Chryseobacterium cucumeris* (99%), respectively. The GenBank accession numbers of the three strains are MK212369, MK212370, and MK212371, respectively. Bacteria belonging to these genera are commonly distributed in soils around the world, including China, India, the USA, and Europe^27–30^. Previously, manganese-oxidizing cultures have been identified as species of *Leptothrix*, *Crenothrix*, *Streptomyces*, and *Hyphomicrobium*^11, 12^. This is the first report of manganese removal by *Streptomyces violarus* and *Chryseobacterium cucumeris*.

*Streptomyces violarus* strain SBP1 (SBP1), *Streptomyces violarus* strain SBP3 (SBP3), and *Chryseobacterium cucumeris* strain SBP7 (SBP7) were investigated for manganese removal efficiency and bacterial growth (as mixed liquor suspended solids (MLSS)) in synthetic groundwater. After 36 h of the experiment (at an initial manganese concentration of 5 mg L^−1^ and MLSS of 10.61–23.31 mg L^−1^), SBP1 provided the highest manganese removal (45.05 ± 2.11%) and bacterial growth (as MLSS) (163.33 ± 6.70 mg L^−1^), while SBP3 and SBP7 gave manganese removal efficiencies of 41.70 ± 1.02 and 9.23 ± 0.60%, respectively.

### Bacterial growth and metal removal by the selected isolate

**Figure 2:**
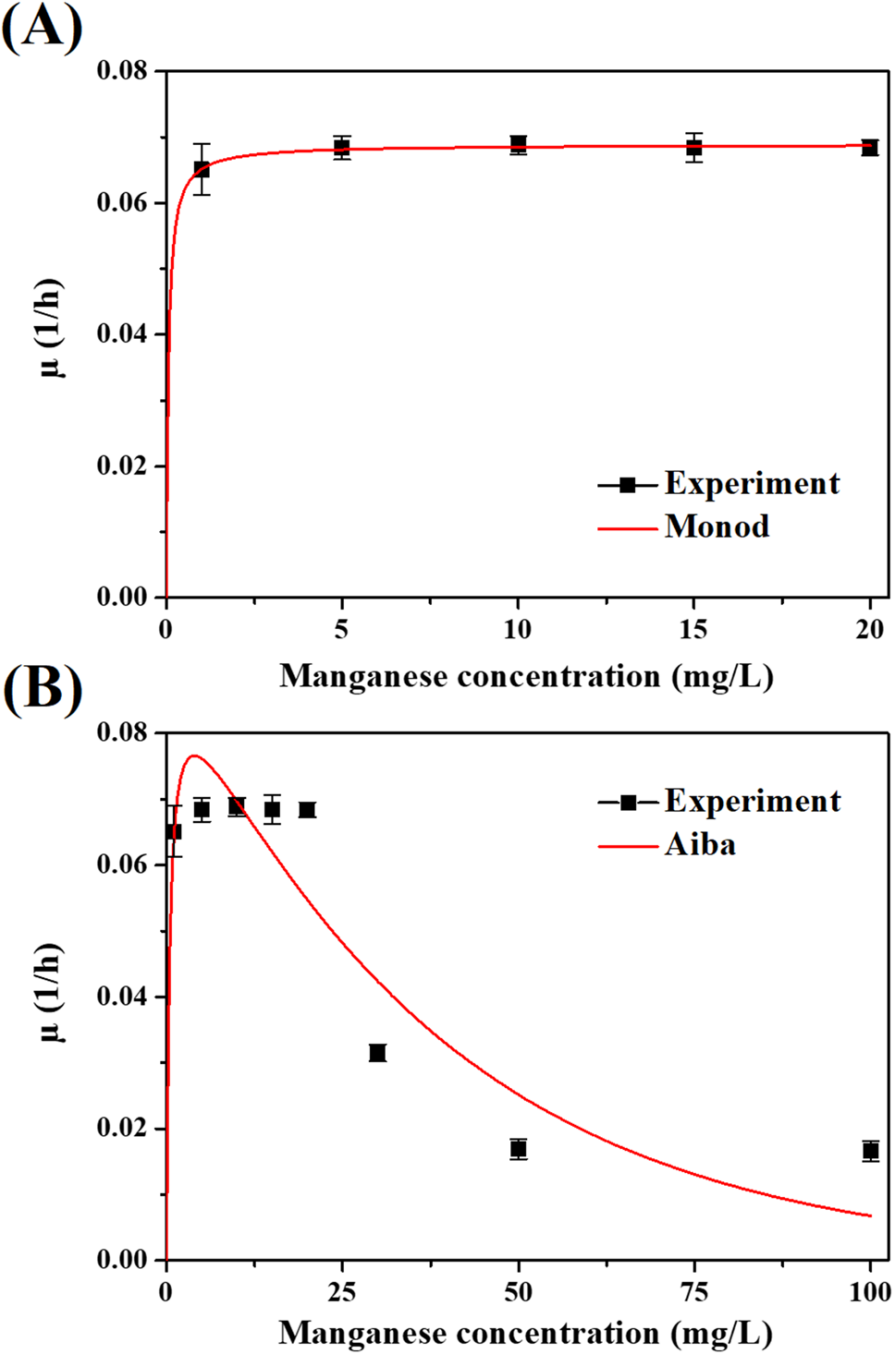
Bacterial growth kinetic experiments: (**A**) predicted Monod model (**B**) predicted Aiba model.

The bacterial growth kinetics of the selected isolate, *Streptomyces violarus* strain SBP1 (SBP1), was estimated following the Monod model. Manganese removal efficiencies and bacterial growth under initial manganese concentrations of 1 to 100 mg L^−1^ are presented in the supplementary material (Fig. S1). For the manganese concentrations of 1, 5, 10, 15, and 20 mg L^−1^, the kinetic coefficients were fit using a Lineweaver–Burk plot with R^2^ = 0.969 (Fig. 2A); µ_max_ of 0.069 h^−1^ and K_s_ of 0.057 mg L^−1^ were estimated. Previously, two consortia enriched from biofilters were observed to remove manganese with µ_max_ 0.017 and 0.050 h^−1^ and K_s_ 0.030 and 0.313 mg L^−1^, respectively^31, 32^. This indicates that the selected isolate from this study grew well in the manganese-contaminated environment, resulting in high µ_max_.

During the experiments with higher manganese concentrations (30–100 mg L^−1^), self-substrate inhibition took place. This result is consistent with prior works about manganese toxicity^7, 10^. The estimations of the self-substrate inhibition kinetic models, including the Haldane, Andrews, Edwards, Aiba, and Yano models, are presented in the supplementary material (Tables S1, S2 and Fig. S2). Among the models, the Aiba model gave the best fit, with a µ_max_, K_s_, and inhibitory constant (K_i_) of 0.095 h^−1^, 0.453 mg L^−1^, and 37.975 mg L^−1^, respectively (Fig. 2B).

Metal removal by SBP1 was demonstrated in both synthetic and natural groundwater. For the synthetic groundwater, SBP1 provided manganese, iron, and arsenic removal efficiencies of 45.93, 81.21, and 38.11%, respectively (metal removal efficiencies are shown in supplementary material, Tables S5). Typically, natural metal ions, cations, and anions may influence the bacterial growth and manganese oxidizing activity leading to impact on manganese removal efficiency^33^. Anions, such as bicarbonate and acetate, could affect metal binding and oxidation^34^. In this study, two natural groundwater sources containing different ions, such as Ca^2+^ and Mg^2+^ were applied. It was found that SBP1 could remove manganese (up to 23.10%) (manganese removal efficiencies shown in supplementary material, Tables S6). Even for the selected natural groundwater with high hardness (790 mg L^−1^ as CaCO_3_) and low dissolved organic carbon contents (0.43 mg L^−1^), SBP1 achieved manganese removal. This result demonstrates that SBP1 could survive and remove manganese in the real environment. The result from this study initially showed the influence of ions. Complete investigation on effects of cations and/or anions on bacterial growth and manganese removal should be further examined.

### Manganese removal mechanism by the selected isolate

#### SEM–EDS analysis

**Figure 3:**
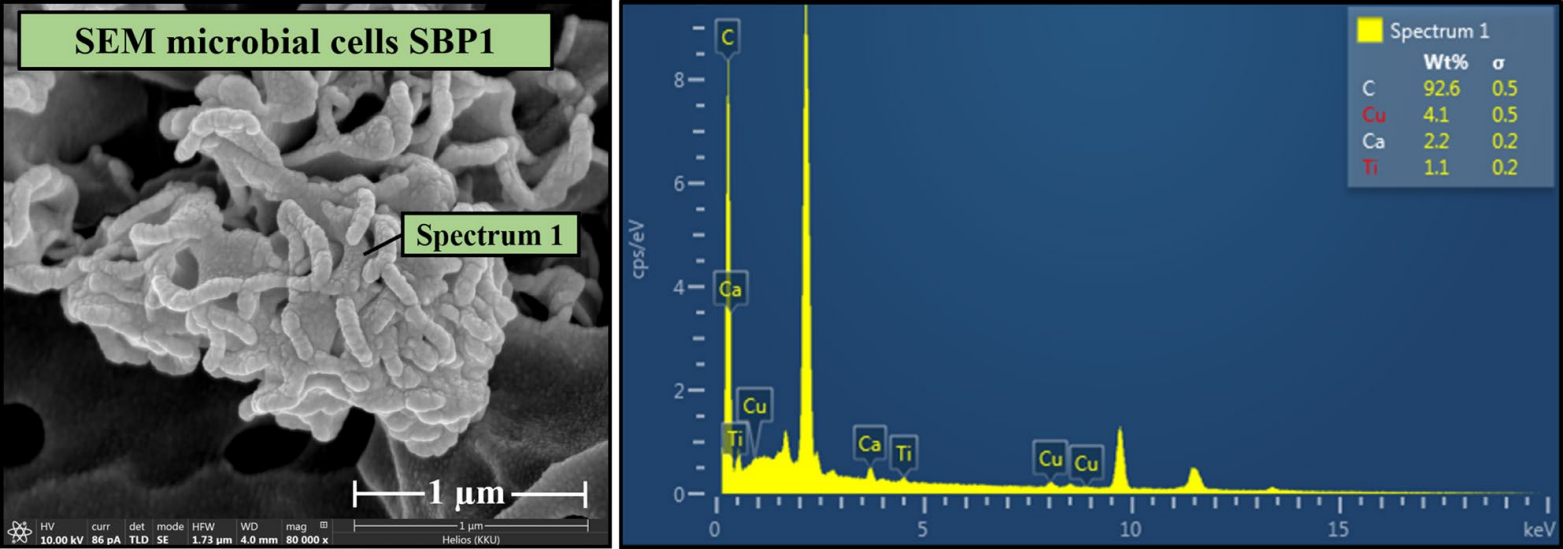
SEM–EDS images: (left) morphology of SBP1 and (right) EDS spectra observed on SBP1.

The bacterium SBP1 was characterised by SEM–EDS. SBP1 is rod-shaped and approximately 0.1 µm in width and 0.4 µm in length (Fig. 3). After the 2-d manganese removal experiment, morphology of SBP1 did not change. The bacterial sample was observed using EDS with the aim of detecting manganese attached to the bacterial cells. No manganese was observed (Fig. 3). This result contrasts with previous work that identified manganese adsorption (60%) based on EDS observation of *Serratia marcescens* in an initial manganese concentration as high as 40 mg L^−1^^25^. In this study, however, a lower manganese concentration (5 mg L^−1^) was applied to simulate contamination conditions in the field. Based on EDS, it is inconclusive whether manganese adsorption on the cell surface took place. Advanced measurement was thus required for determination of the manganese removal mechanism.

#### XPS and XAS techniques

**Figure 4:**
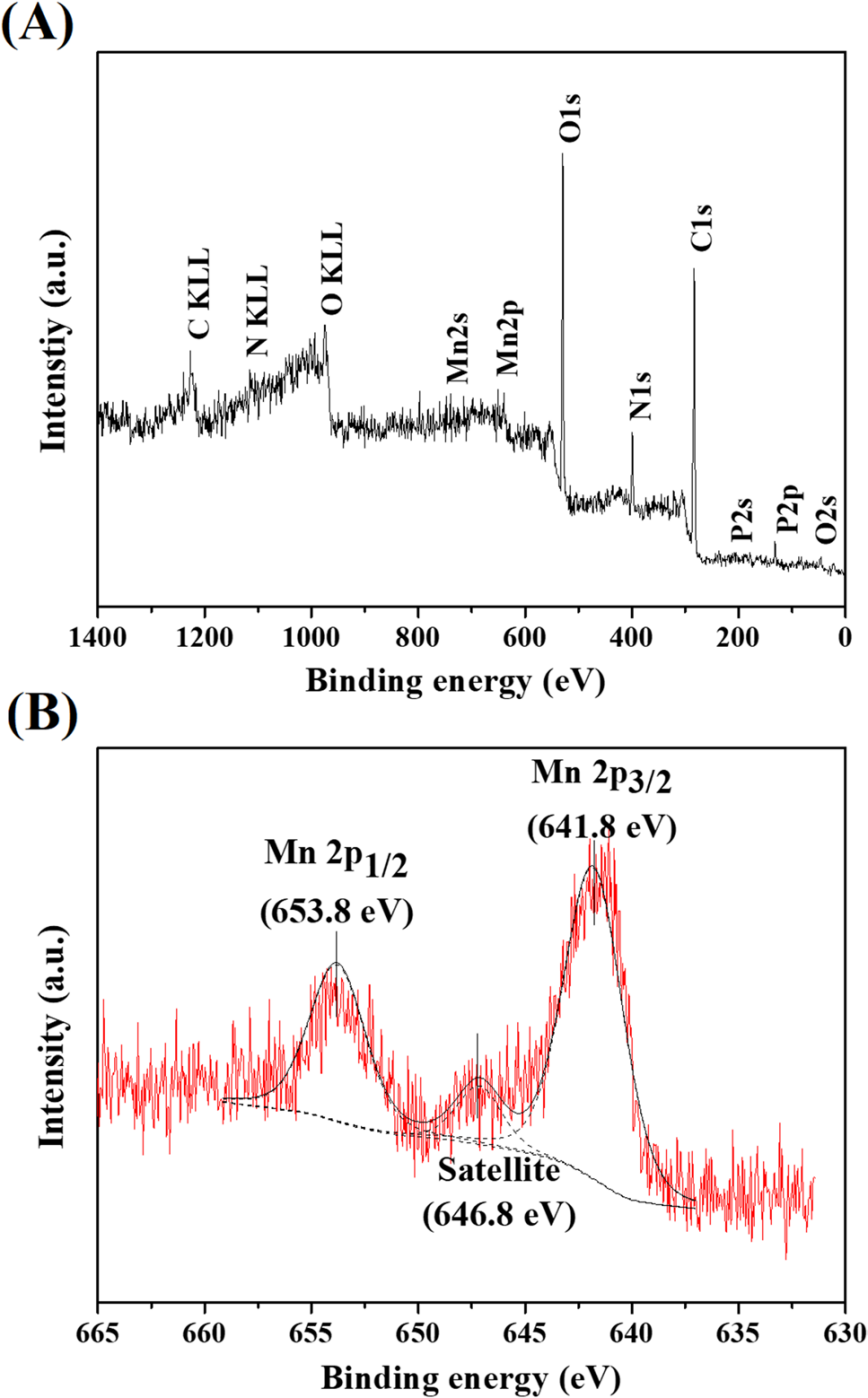
XPS spectra of SBP1 (after manganese oxidation experiment): (**A**) survey scan and (**B**) high resolution spectrum of Mn2p peak.

The XPS technique was performed to detect manganese and determine its oxidation state on the surface of the cell sample after the manganese removal experiment. The XPS survey spectrum is presented in Fig. 4A. Oxygen, nitrogen, phosphorus, and carbon were found in the sample. The high resolution of the XPS spectrum of Mn2p peaks in Fig. 4B includes two major distinct peaks at binding energies of 641.8 and 653.8 eV, which correspond to Mn2p_3/2_ and Mn2p_1/2_, respectively. Along with the Mn2p_3/2_, the shake-up satellite peak at 646.6 eV was also observed. The observed satellite feature at 646.6 eV is only present for MnO, representing Mn^2+^.

**Figure 5:**
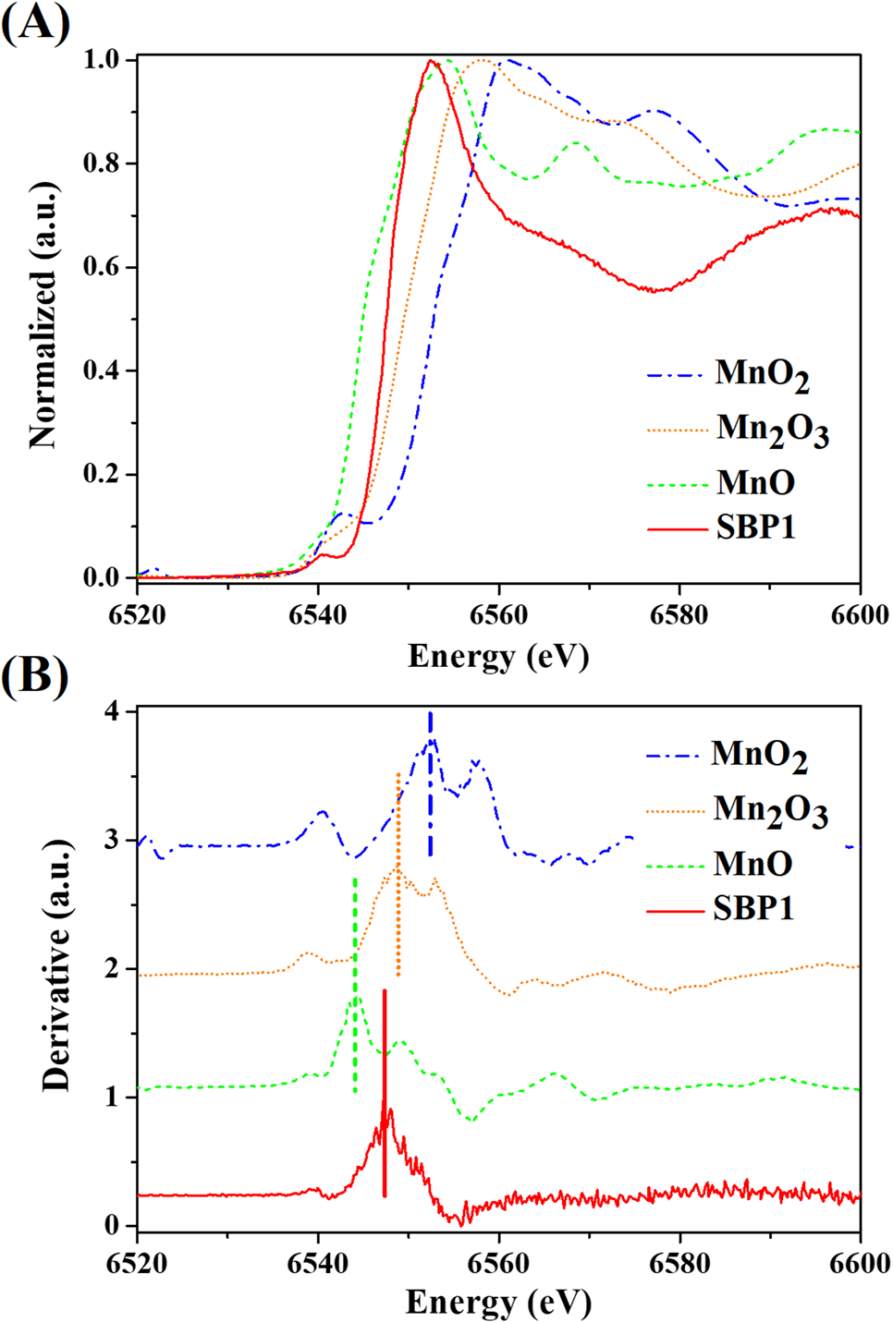
Manganese K-edge XANES data of SBP1 (after manganese oxidation experiment), MnO, Mn_2_O_3_, and MnO_2_: (**A**) normalized and (**B**) derivative spectra.

SBP1 was also characterised after the manganese removal experiment by X-ray Absorption Near Edge Structure (XANES) and Extended X-ray Absorption Fine Structure (EXAFS) to investigate manganese valence state and local structure. Figure 5A shows the normalized XANES spectra at the manganese K-edge of the SBP1 sample compared with manganese standards, including MnO, Mn_2_O_3_, and MnO_2_, referred to as Mn^2+^, Mn^3+^, and Mn^4+^, respectively. Figure 5A reveals the different positions of absorption edge energy for the references and sample. To compare absorption edge energy among the sample and references, the derivative of the XANES spectra was calculated (Fig. 5B). The absorption edge energy of the SBP1 sample was between those of MnO and Mn_2_O_3_. This indicates that the oxidation state of SBP1 comprises Mn^2+^ and Mn^3+^. The local structure around manganese atoms in the SBP1 sample was investigated by EXAFS, as shown in the supplementary material (Fig. S3 and Tables S7). A peak position of the main peak between 1 and 2 Å is consistent with the binding of manganese to oxygen^35^. In this study, the peak position (1.5 Å) of the main peak corresponds to Mn–O bonding. EXAFS fitting revealed that manganese is surrounded by four oxygen atoms at interatomic distances of 2.14 Å.

## Discussion

The results from this study identified numerous manganese-tolerant bacteria in the environment. Typically, manganese is a required element as a co-factor for bacterial cells. It is involved in transcriptional regulation, developmental and metabolic processes, and protection of the bacterial cell against oxidative stress. Previous studies have isolated manganese-oxidizing bacteria from soil and water treatment systems, including filters, water pipes, and sediment in groundwater wells in the USA, China, and South Africa^17–19^. Bacterial cultures including *Pseudomonas putida* strain MnB1, *Leptothrix* spp., *Bacillus* spp., and *Acinetobacter* sp. strain LB1 have been reported based on these studies as effective manganese-oxidizing microbes. SBP1 removed manganese well compared to previously reported cultures (Table 2). In addition, this is the first report on enrichment of manganese-tolerant bacteria in Thailand. The cultures isolated here can withstand a typically toxic environment (manganese concentration of 100 mg L^−1^).

**Table 2:** Biological manganese removal by isolated cultures.

Strain	Source	Country	Manganese concentration during isolation (mg L^−1^)	Removal efficiency	Reference
*Arthrobacter* sp.	Manganese nodule	New York, USA	–	60% at initial manganese 0.15 mg L^−1^	36
*Citrobacter freundii*	Manganese concretion	Peloponnese, Greece	–	50% at initial manganese 0.4 mg L^−1^	37
*Brevibacillus brevis* strain MO1	Activated sludge	Harbin, China	30	65% at initial manganese 5.5 mg L^−1^	38
*Brevibacillus parabrevis* strain MO2	Activated sludge	Harbin, China	30	66% at initial manganese 5.5 mg L^−1^	38
*Bacillus* SG-1	Marine sediment	California, USA	50	65% at initial manganese 55 mg L^−1^	13
*Leptothrix discophora* SS-1	Metallic surface film	New York, USA	100	90% at initial manganese 3 mg L^−1^	14
*Streptomyces violarus* strain SBP1	Soil	Khon Kaen, Thailand	100	45% at initial managanese 5 mg L^−1^	This study
*Streptomyces violarus* strain SBP2	Soil	Khon Kaen, Thailand	100	41% at initial managanese 5 mg L^−1^	This study
*Chryseobacterium cucumeris* strain SBP7	Soil	Khon Kaen, Thailand	100	9% at initial managanese 5 mg L^−1^	This study

The manganese removal performance of the selected isolate (SBP1) presented in this study was comparable to those previously reported for manganese-oxidizing cultures. Adams and Ghiorse^14^ reported manganese removal by *Leptothrix discophora* of approximately 90% in 24 h (initial manganese concentration of 3 mg L^−1^). Recently, Zhao et al.^38^ found manganese removal efficiencies for *Brevibacillus brevis* MO1 and *Brevibacillus parabrevis* MO2 of approximately 40–65% during 10-d experiments with an initial manganese concentration of 5.5 mg L^−1^.

This bacterium, SBP1, was previously investigated for manganese removal from synthetic groundwater with low organic carbon supplement^39^. The previous report found lower biological manganese removal performance (efficiency of up to 11%). The main removal mechanism reported earlier was adsorption by biochar. The result correlated well with the result for natural groundwater (low organic carbon). This indicates that the organic carbon content plays an important role in manganese removal by SBP1. Moreover, SBP1 can remove iron and arsenic. This correlates well with prior works^11, 40^. Manganese-oxidizing bacteria including strains in genera *Leptothrix*, *Crenothrix*, and *Metallogenium* have successfully removed iron and manganese in previous studies. This is because the bacteria contain enzymes related to metal oxidation. Along with manganese and iron bio-oxidation, arsenic removal has also been reported^41^. The results presented in this study indicate that SBP1 effectively removed manganese, iron, and arsenic. Further work on the metal removal mechanism of SBP1 should be performed.

With respect to the manganese removal kinetics of SBP1, previous studies have also reported that self-substrate inhibition kinetics followed the Aiba model, such as studies of alcohol fermentation, ammonia oxidation, and benzene degradation^42–44^. This study is the first report on manganese biotransformation. It could be stated that the Aiba model fits well for a wide range of substrates and microbial cultures.

The manganese removal mechanism could be described by the synchrotron-based analysis. The XPS result (Fig. 4A) represents the typical composition of microbial cells^24^. Manganese was also observed on the surface of the sample after the manganese removal experiment. The observed satellite peak at 646.6 eV (Fig. 4B) indicates the presence of manganese (Mn^2+^) on the cell surface^23^. The bacterial adsorption mechanism is consistent with previous findings where living and dead cells were tested for manganese bio-sorption^24^.

The XANES result confirmed that the manganese removal by SBP1 occurred via adsorption and biotransformation, resulting in the observation of Mn^2+^ and Mn^3+^. In addition, the ratio of Mn^2+^ to Mn^3+^ was calculated based on absorption edge energy. SBP1 comprised Mn^2+^ and Mn^3+^ at 20% and 80%, respectively. Normally, microorganisms are known to be a natural sorbent in the environment^24^. In this work, the proportions of Mn^2+^ and Mn^3+^ based on the XANES result identified manganese biotransformation by SBP1 as a major manganese removal mechanism. Mn^3+^ is not stable in the environment and may convert to Mn^2+^ or Mn^4+^^32^. During the experiment, Mn^2+^ accumulation was not observed; Mn^3+^ would be further oxidized into Mn^4+^ later on.

The advanced spectroscopic techniques clearly indicated that SBP1 successfully removed manganese from contaminated water via oxidation (80%) and adsorption (20%) processes. Theoretically, the physicochemical manganese removal from contaminated water is a two-step process. Firstly, soluble manganese (Mn^2+^) is oxidized to particulate manganese (Mn^3+^ or Mn^4+^) via chemical oxidation or aeration. Then, the particle was separated in water filtration unit. In practice, the chemical oxidation (or aeration) did not well transform manganese leading to soluble manganese transport through the filter and presence in the treated water. The result from this study could state potential of SBP1 for enhancement of manganese removal in filtration unit. The isolated culture could apply in a filter as an inoculant to transform soluble manganese to be particulate manganese. Then, the particle (oxidized manganese) is removed from filter bed by back-washing process.

Overall results from this study could preliminarily describe manganese removal mechanism for SBP1. Manganese adsorption occurred at the cell surface. Oxidation was the main process; however, complete oxidation (formation of Mn^4+^) did not take place. This incomplete oxidation issue could be governed by manganese-oxidizing activity of SBP1. Manganese oxidation by bacteria is typically from two enzymes including multicopper oxidases or peroxidase cyclooxygenases^33, 45, 46^. Multicopper oxidases have been widely identified in numerous bacterial species^33, 46, 47^. The enzymes gave different manganese-oxidizing activities under different environmental conditions, such as presence of metal ions or metal chelators (such as o-phenanthroline and EDTA)^33^. For better clarification, manganese-oxidizing activity of SBP1 should be further investigated. The further investigation could be applied for enhancement of manganese-oxidizing activity.

## Conclusions

Twenty-one pure bacterial strains were isolated from manganese-contaminated sites. Isolate SBP1 provided the greatest manganese removal (46%). The isolate also removed iron and arsenic. During manganese removal experiments under initial concentrations of greater than 30 mg L^−1^, growth of SBP1 was inhibited. Self-substrate inhibition kinetics followed the Aiba model. Using synchrotron techniques, Mn^2+^ was observed on the cell surface. The XANES result showed a mixture of Mn^2+^ and Mn^3+^, which indicates a combination of manganese adsorption and bio-oxidation by SBP1. SBP1 shows promise for future application as an augmented microbial culture for biological manganese removal.

## Methods

### Bacterial medium and synthetic groundwater

For preliminary screening, a bacterial medium was modified from Cerrato et al.^19^ The medium (pH of 6.8) contained 0.308 g L^−1^ (or 0.0154 g L^−1^ for long-term cultivation) of MnSO_4_·H_2_O, 0.001 g L^−1^ of FeSO_4_·7H_2_O, 2.383 g L^−1^ of HEPES buffer, 1 g L^−1^ of peptone, and 0.25 g L^−1^ of yeast extract. The medium for bacterial isolation and long-term cultivation provided 100 and 5 mg manganese L^−1^, respectively. For a solid medium, agar (1.5% w/v) was added.

The synthetic groundwater contained 0.0001 g L^−1^ of K_2_HPO_4_, 0.01 g L^−1^ of Na_2_SO_4_, 0.008 g L^−1^ of NaHCO_3_, 0.0154 g L^−1^ of MnSO_4_·H_2_O, 0.05 mg L^−1^ of FeSO_4_·7H_2_O, 0.67 mg L^−1^ of CaCl_2_·2H_2_O, 0.02 g L^−1^ of MgSO_4_·7H_2_O, 0.002 g L^−1^ of NH_4_Cl, 1 g L^−1^ of peptone, and 0.25 g L^−1^ of yeast extract. For the experiments demonstrating removal of other metals, the synthetic groundwater contained similar compositions with the addition of 1 and 5 mg L^−1^ of iron or arsenic (more information in supplementary material). All chemicals were purchased from RCI labscan (Thailand), Hi-media (India), Ajax finechem (Australia), QReC (New Zealand), and Sigma-Aldrich (Singapore) via local chemical suppliers.

### Manganese-oxidizing bacterial enrichment and isolation

Samples of soil and filter medium that had experienced manganese contamination were collected from Ban Phai district (16°4′42″N, 102°38′40″E) and Kranuan district (16°4′42″N, 102°38′40″E), Khon Kaen, Thailand. The soil and filter medium samples were air-dried at room temperature overnight. Then, 10 g of each air-dried sample was inoculated in 100 mL of bacterial medium. The samples were incubated at room temperature on an orbital shaker at 150 rpm for 1 week. The samples were then sub-cultured into fresh medium 6 times to obtain stable mixed cultures. Bacterial cultures were isolated using spread and streak plate techniques.

The isolated bacteria were evaluated for (1) manganese oxidation potential using the leucoberbelin blue (LBB) method and (2) manganese removal efficiency. It is noted that the LBB assay was performed in the solid medium, while manganese removal efficiency was carried out in the liquid medium. The LBB method was applied to distinguish dissolved manganese and oxidized manganese (Mn^3+^ and Mn^4+^)^48^. The LBB reagent (0.04% (w/v) in 10 mM acetic acid) was prepared and dropped on isolated colonies in agar medium. Then, the isolated colonies were incubated in the dark at room temperature for 1 h. The agar medium turned from colourless to blue in the presence of oxidized manganese (positive). Five replicates were performed to confirm manganese oxidation^26^. The isolates with positive results from the LBB method were selected for the subsequent experiments.

Triplicate manganese removal experiments were performed using the selected isolates. For each isolate, 10 mL was inoculated in the bacterial medium with an initial manganese concentration of 5 mg L^−1^. The isolates were shaken at 150 rpm and maintained at room temperature for 168 h. The manganese concentration in the water samples was then analysed. The manganese removal efficiency was calculated using the equation shown below.

1 $${\text{Manganese removal efficiency}} (\% ) = \frac{{{\text{Mn}}_{i} - {\text{Mn}}_{r} }}{{{\text{Mn}}_{i} }} \times 100,$$

where Mn_i_ and Mn_r_ are the initial and remaining manganese concentrations (mg L^−1^), respectively. The isolates with the three highest removal efficiencies were selected for further experiments.

### Manganese-oxidizing bacteria selection

Three bacterial isolates from the previous section were identified by their 16S rRNA genes. The isolates were cultivated in the bacterial medium agar for 2 days. The samples were sent for 16S rRNA gene identification (Macrogen, Korea). The full-length 16S rRNA gene sequences were amplified using polymerase chain reaction amplification with two universal primers (27f: 5ʹ-AGA GTT TGA TCM TGG CTC AG and 1492r: 5ʹ-TAC GGY TAC CTT GTT ACG ACT T), aligned using BioEdit 7.2.6, and compared to sequences from the NCBI BLAST GenBank nucleotide sequence database. A phylogenetic tree of the three isolates compared to other related sequences was constructed. The maximum likelihood method based on the Tamura-Nei model was applied using MEGA7^49^. The tree with the highest log likelihood (− 9,324.80) was applied. There were a total of 1,217 positions in the final dataset.

The three isolates were investigated for their manganese removal performance and growth in synthetic groundwater. For the manganese removal performance test (triplicate experiments), the isolates were inoculated in the synthetic groundwater (10% inoculation) with an initial manganese concentration of 5 mg L^−1^. Initial cell numbers of approximately 10^4^ CFU mL^−1^ (equal to MLSS of 100–200 mg L^−1^) were applied. The isolates were shaken at 150 rpm at room temperature for 48 h. The manganese concentrations in the water samples were then analysed. The manganese removal efficiency was calculated following Eq. 1 provided earlier. The isolate with the highest removal efficiency was selected for the kinetic growth experiment.

### Bacterial growth and metal removal

Triplicate experiments were performed to investigate bacterial growth and metal removal. The selected bacterium (10 mL) was inoculated in 100-mL aliquots of synthetic groundwater with manganese concentrations of 1, 5, 10, 15, 20, 30, 50, and 100 mg L^−1^. The reactors with the bacterium were then shaken at 150 rpm at room temperature for 48 h. Water samples were collected at 0, 12, 24, 36, and 48 h. Bacterial cells were measured as MLSS. Specific growth rates were calculated based on bacterial cell data. The bacterial growth kinetics (µ) were then estimated following the Monod model. The kinetic parameters were replotted and calculated using a Lineweaver–Burk plot. The specific growth rate, Monod model, and Lineweaver–Burk plot equations were as follows:

2 $$\frac{{{\text{dX}}}}{{\text{X}}}= \mu {\text{dt,}}$$

3 $$\mu  = \mu _{{\text{max}}}\frac{{\text{S}}}{{{\text{K}}_{{\text{S}}} {\text{ } + \text{ S}}}},$$

4 $$\frac{{1}}{\mu } = \frac{{{\text{K}}_{{\text{S}}} }}{{\mu_{{\max}} }}{ }\frac{{1}}{{\text{S}}}{ + }\frac{{1}}{{\mu_{{\max}} }},$$

where X is bacterial cell concentration (mg-MLSS L^−1^), t is time (h), µ is specific growth rate (h^−1^), µ_max_ is maximum specific growth rate (h^−1^), S is manganese concentration (mg L^−1^), and K_s_ is half-velocity constant (mg L^−1^). Alternatively, the self-substrate inhibition kinetics were estimated following the Haldane, Andrews, Edwards, Aiba, and Yano models (based on the microbial growth rates under different initial manganese concentrations)^50^.

To quantify the metal removal performance of the selected isolate, metal removal experiments were performed. The experiments of manganese, iron, and arsenic removal were tested using 100 mg-MLSS L^−1^ of the selected isolate at initial metal concentrations of 1 and 5 mg L^−1^ in the synthetic groundwater (synthetic groundwater formulation shown in supplementary material, Table S3). For the removal of manganese from natural groundwater by the selected isolate, natural groundwaters with different characteristics (from two locations) were obtained. The groundwater characteristics and information are shown in the supplementary material, Table S4. The selected isolates of 200 or 500 mg-MLSS L^−1^ were inoculated. The batch reactors were shaken at 150 rpm and room temperature for 48 h. The metal removal efficiency percentage was then calculated.

### Microscopic and synchrotron-based spectroscopic techniques

Microscopic and synchrotron-based spectroscopic techniques including SEM–EDS, XPS, and XAS were used to characterise the bacterial cells to elucidate the mechanism of manganese removal. Bacterial samples were collected before and after the manganese removal experiment performed with a manganese concentration of 5 mg L^−1^. SEM–EDS was used to investigate microbial cell morphology and elemental composition in the samples. The samples were also observed using a field emission scanning electron microscope coupled with a focused gallium ion beam (FIB-FESEM) (Thermo Fisher, USA). The bacterial cell samples were dehydrated followed Taweetanawanit et al^51^.

The XPS technique was used to study the chemical composition and confirm the oxidation state of elements on the bacterial surface. The XPS measurement was carried out using a PHI5000 Versa Probe II (ULVAC-PHI, Japan) at the SUT-NANOTEC-SLRI Joint Research Facility (SLRI, Thailand). To investigate the oxidation state of elements and species of neighbour atoms for the entire bacterial cells, XAS techniques were selected: XANES and EXAFS, respectively. Manganese K-edge XANES and EXAFS were detected on BL5.2: SUT-NANOTEC-SLRI XAS Beamline SLRI, Thailand^52, 53^. The bacterium sample for XPS and XAS was taken at 2 d, then filtered on cellulose acetate membrane (0.45 µm, Filtrex, USA) and dried in an oven at 70 °C for 1.5 h.

### Analytical procedures

Manganese analysis using nitric acid digestion was performed following standard method 3030E^54^. After filtering a 25 mL water sample using a nylon filter (0.22 µm, Agela Technologies, USA), the sample was digested on a hot plate in a fume hood until reaching a sample volume of 5 mL. The digested sample was mixed with 10 mL concentrated nitric acid (RCI labscan, Thailand). Then, the mixture was boiled until reaching a sample volume of 5 mL. The final 5-mL sample was adjusted to 25 mL by adding deionized water. The digested sample was analysed using an Atomic Absorption Spectrophotometer (AAS) (AAnalyst 800, Perkin Elmer, Singapore).

The bacterial cells (as MLSS) were measured using the gravimetric method following standard method 2540D^54^. Water samples of 100 mL were filtered through a GF/C glass microfiber filter (1.2 µm, Whatman, UK). The filtered sample was dried in an oven at 105 °C for 1.5 h.

## Supplementary information

Supplementary file1
