# Supplementary material for: Manganese-contaminated groundwater treatment by novel bacterial isolates: kinetic study and mechanism analysis using synchrotron-based techniques
Source: Sci Rep. 2020 Aug 7;10:13391. doi: 10.1038/s41598-020-70355-w (PMC7414890; doi:10.1038/s41598-020-70355-w)
Supplement: Supplementary file 1 — Supplementary file1 [file 41598_2020_70355_MOESM1_ESM.pdf]

Supplementary Material

Manganese-contaminated Groundwater Treatment by Novel Bacterial Isolates: Kinetic Study and Mechanism Analysis Using Synchrotron-based Techniques

Nakharin Therdkiattikul[1], Thunyalux Ratpukdi[1,2], Pinit Kidkhunthod[3], Narong Chanlek[3], Sumana Siripattanakul-Ratpukdi[1,2*]

[1] Department of Environmental Engineering, Faculty of Engineering and Research Center for Environmental and Hazardous Substance Management, Khon Kaen University, Khon Kaen 40002, Thailand

[2] Center of Excellence on Hazardous Substance Management (HSM), Bangkok 10330, Thailand

[3] Synchrotron Light Research Institute (Public Organization), Nakhon Ratchasima 30000, Thailand

* e-mail: sumana.r@kku.ac.th

Manganese removal and bacterial growth of SBP1 in synthetic groundwater

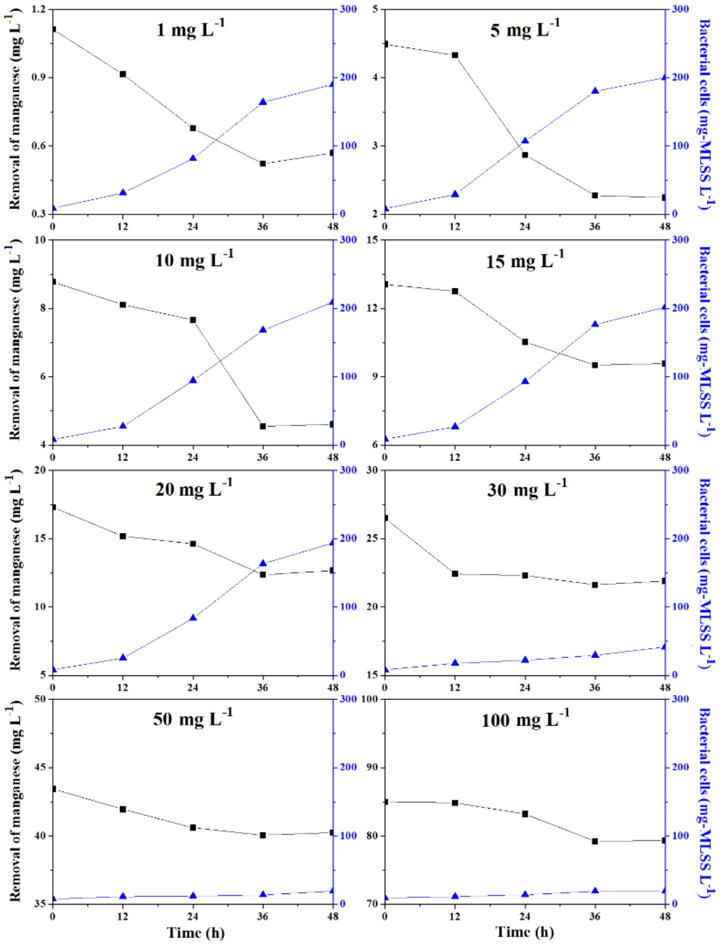

Figure S1. Manganese removal and bacterial cells of SBP1

Estimation of self-substrate inhibition kinetics

The self-substrate inhibition kinetics was estimated followed different mathematic models as shown in Table S1. The experimental results were fitted and estimated the kinetic parameters using non-linear regression method (Generalized reduced gradient methodology) through Solver function in MS Excel (Fig. S2). The kinetic parameters and root mean square error (RMSE) were shown in Table S2. Based on RSME values, the self-substrate inhibition kinetics in this study followed Aiba model.

Table S1 Self-substrate inhibition kinetic equations applied in this study

Model	Equation	Reference
Haldane	$\mu = \dfrac{\mu_{max} S}{(S + (\frac{S^2}{K_i}) + K_s + (\frac{S K_s}{K_i}))}$	1
Andrews	$\mu = \dfrac{\mu_{max}}{(1 + (\frac{K_s}{S}) + (S + K_i))}$	2
Edwards	$\mu = \mu_{max} (\exp^{-(\frac{S}{K_i})} - \exp^{-(\frac{S}{K_s})})$	3
Aiba	$\mu = \dfrac{\mu_{max} S}{(S + K_s)} (\exp^{-(\frac{S}{K_i})})$	4
Yano	$\mu = \dfrac{\mu_{max} S}{(S + K_s + (\frac{S^2}{K_i}) + (\frac{S}{K}))}$	5

μ = Predicted specific growth rate (h^{-1}); μ_{max} = Predicted maximum specific growth rate (h^{-1}); S = Substrate concentration (mg L^{-1}); Ks = Half-velocity constant (mg L^{-1}); Ki = Substrate inhibition constant (mg L^{-1}); K = Yano constant.

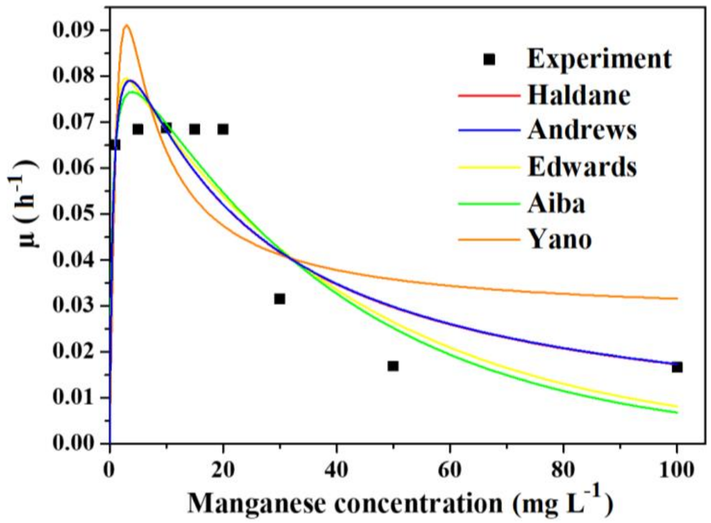

Figure S2. Experiment and modelling results of manganese biotransformation by SBP1

Table S2 Predicted kinetic parameter and RSME values

**Model**	**Kinetic parameters**				**RMSE**
	**μ_{max} (h^{-1})**	**K_s (mg L^{-1})**	**K_i (mg L^{-1})**	**K (mg L^{-1})**	
Haldane	0.113	0.701	18.091	-	0.0095
Andrews	0.109	0.668	19.005	-	0.0094
Edwards	0.086	0.671	42.257	-	0.0088
**Aiba**	**0.095**	**0.453**	**37.975**	**-**	**0.0084**
Yano	0.273	6.028	1.707	17.123	0.0148

Demonstration of manganese, iron, and arsenic removal in synthetic groundwater and manganese removal in natural groundwater

Groundwater characteristics and information

Table S3 Synthetic groundwater formulation for manganese, iron, and arsenic removal

Test	Metal	Synthetic groundwater composition
1	Manganese 1 mg L^{-1}	**$MnSO_4 \cdot H_2O$ 0.0031 g L^{-1}**, K_2HPO_4 0.0001 g L^{-1}, Na_2SO_4 0.01 g L^{-1}, $NaHCO_3$ 0.008 g L^{-1}, $CaCl_2 \cdot 2H_2O$ 0.00067 g L^{-1}, $MgSO_4 \cdot 7H_2O$ 0.02 g L^{-1}, NH_4Cl 0.002 g L^{-1}, peptone 1 g L^{-1}, and yeast extract 0.25 g L^{-1}
2	Manganese 5 mg L^{-1}	**$MnSO_4 \cdot H_2O$ 0.0154 g L^{-1}**, K_2HPO_4 0.0001 g L^{-1}, Na_2SO_4 0.01 g L^{-1}, $NaHCO_3$ 0.008 g L^{-1}, $CaCl_2 \cdot 2H_2O$ 0.00067 g L^{-1}, $MgSO_4 \cdot 7H_2O$ 0.02 g L^{-1}, NH_4Cl 0.002 g L^{-1}, peptone 1 g L^{-1}, and yeast extract 0.25 g L^{-1}
3	Iron 1 mg L^{-1}	**$FeSO_4 \cdot 7H_2O$ 0.0050 g L^{-1}**, K_2HPO_4 0.0001 g L^{-1}, Na_2SO_4 0.01 g L^{-1}, $NaHCO_3$ 0.008 g L^{-1}, $CaCl_2 \cdot 2H_2O$ 0.00067 g L^{-1}, $MgSO_4 \cdot 7H_2O$ 0.02 g L^{-1}, NH_4Cl 0.002 g L^{-1}, peptone 1 g L^{-1}, and yeast extract 0.25 g L^{-1}
4	Iron 5 mg L^{-1}	**$FeSO_4 \cdot 7H_2O$ 0.0250 g L^{-1}**, K_2HPO_4 0.0001 g L^{-1}, Na_2SO_4 0.01 g L^{-1}, $NaHCO_3$ 0.008 g L^{-1}, $CaCl_2 \cdot 2H_2O$ 0.00067 g L^{-1}, $MgSO_4 \cdot 7H_2O$ 0.02 g L^{-1}, NH_4Cl 0.002 g L^{-1}, peptone 1 g L^{-1}, and yeast extract 0.25 g L^{-1}
5	Arsenic 1 mg L^{-1}	**$NaAsO_2$ 0.0017 g L^{-1}**, K_2HPO_4 0.0001 g L^{-1}, Na_2SO_4 0.01 g L^{-1}, $NaHCO_3$ 0.008 g L^{-1}, $CaCl_2 \cdot 2H_2O$ 0.00067 g L^{-1}, $MgSO_4 \cdot 7H_2O$ 0.02 g L^{-1}, NH_4Cl 0.002 g L^{-1}, peptone 1 g L^{-1}, and yeast extract 0.25 g L^{-1}
6	Arsenic 5 mg L^{-1}	**$NaAsO_2$ 0.0087 g L^{-1}**, K_2HPO_4 0.0001 g L^{-1}, Na_2SO_4 0.01 g L^{-1}, $NaHCO_3$ 0.008 g L^{-1}, $CaCl_2 \cdot 2H_2O$ 0.00067 g L^{-1}, $MgSO_4 \cdot 7H_2O$ 0.02 g L^{-1}, NH_4Cl 0.002 g L^{-1}, peptone 1 g L^{-1}, and yeast extract 0.25 g L^{-1}

Table S4 Water characteristics and location information of natural groundwaters

Water information / characteristics	Groundwater sources	
	BH1	BH2
Location	Ban Phai, Khon Kaen, Thailand	Mueang, Khon Kaen, Thailand
Geographic coordinate	16°4'42"N, 102°38'40"E	16°30'16"N, 102°47'17"E
Use	Raw water for industrial factory	Water for gardening
Manganese (mg L^{-1})	0.43	1.08 (adding $MnSO_4{\cdot}H_2O$)
Iron (mg L^{-1})	<0.1	0.22
Arsenic (mg L^{-1})	<0.05	<0.05
Total hardness (mg L^{-1} as $CaCO_3$)	790.00	128.50
Calcium (mg L^{-1})	210.22	41.08
Magnesium (mg L^{-1})	64.52	6.32
pH	6.76	7.54
DO (mg L^{-1})	6.34	7.32
ORP (mV)	140.70	88.80
EC (mS cm^{-1})	4.32	1.23
DOC (mg L^{-1})	0.66	0.43

Water quality analytical procedure

Natural groundwater samples were examined the water quality. pH, DO, ORP, and EC were analyzed by electrometric method using a multi-meter (HQ40d, HACH, USA). Total hardness, calcium, and magnesium were evaluated following standard method of EDTA titrimetric method 2340, EDTA titrimethic method 3500-Ca, and calculation method 3500-Mg, respectively.[6] Dissolved organic carbon in natural groundwater was performed. The water sample was filtrated by nylon filter (0.45 µm). Filtrated water was analyzed using a total organic carbon analyzer (TOC multi N/C 2100, Analytic Jena, Germany).

For metal analysis from synthetic and natural groundwater samples, nitric acid digestion method was performed following standard method 3030E.[6] After filtering a 25 mL water sample using a nylon filter (0.22 µm), the sample was digested on a hot plate in a fume hood until reaching a sample volume of 5 mL. The digested sample was mixed with 10 mL nitric acid (conc.) (RCI labscan, Thailand). Then, the mixture was boiled until reaching a sample volume of 5 mL. The final 5-mL sample was adjusted to 25 mL by adding deionized water. The metal concentration in synthetic groundwater was analyzed using an Inductively Coupled Plasma-Optical Emission Spectrometry (ICP-OES) (Optima 2100DV, Perkin Elmer, Singapore). The metal remaining in the natural groundwater was detected using Atomic Absorption Spectrophotometer (AAS) (PinAAcle 900F, Perkin Elmer, Germany).

Metal removal efficiencies

Table S5 Metal removal efficiency by SBP1 in the synthetic groundwater

Test	Metal	Initial metal concentration (mg L^{-1})	Metal removal (%)
1	Manganese	1	45.93 ± 1.28
2	Manganese	5	42.09 ± 1.69
3	Iron	1	81.21 ± 1.99
4	Iron	5	62.22 ± 1.13
5	Arsenic	1	38.11 ± 1.13
6	Arsenic	5	26.52 ± 1.87

Table S6 Manganese removal efficiency by SBP1 in natural groundwater

Sample	Initial cells (mg-MLSS L^{-1})	Manganese removal at 48 h (%)	
		BH1	BH2
1	200	17.62 ± 0.25	19.64 ± 0.45
2	500	18.06 ± 0.28	23.10 ± 0.22

Mn K-edge EXAFS spectra of SBP1

The Mn K-edge EXAFS spactra and fitting result is shown in Fig. S3. The fitting line was nearby experiment at first shell which indicated Mn-O bonding. The EXAFS fitting result parameters including model coordination number (CN), Debye–Waller factor coefficient (σ^2), energy shift (E_0), fitting residual (R_{eff}), and atomic separation distance (R) was shown in Table S3.

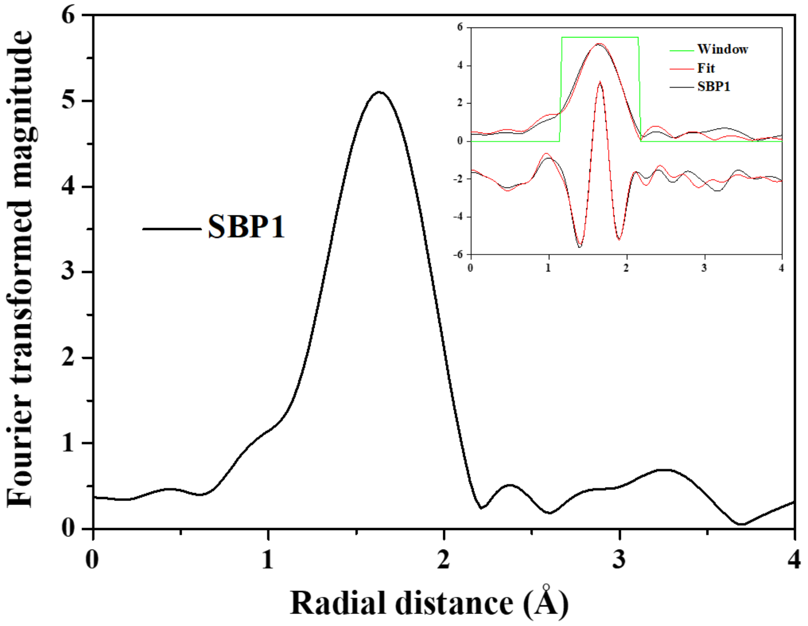

Figure S3. Fourier transform of Mn K-edge EXAFS spectra and fitting result

Table S7 Fitting results of Mn K-edge EXAFS spectra

Name	CN	σ^2	E_0	R_{eff}	R
Mn-O	4.050	0.007	-2.179	2.100	2.142

References

1. Haldane, J.B.S. *Enzymes*, (MIT Press, 1965).
2. Andrews, J.F. A mathematical model for the continuous culture of microorganisms utilizing inhibitory substrates. *Biotechnol. Bioeng.* **10**, 707–723, https://doi.org/10.1002/ bit.260100602 (1968).
3. Edwards, V.H. The influence of high substrate concentrations on microbial kinetics. *Biotechnol. Bioeng.* **12**, 679–712, https://doi.org/10.1002/bit.260120504 (1970).
4. Aiba, S., Shoda, M. & Nagalani, M. Kinetics of product inhibition in alcohol fermentation. *Biotechnol. Bioeng*. **10**, 845–864, https://doi.org/10.1002/bit.260100610 (1968).
5. Yano, T., Nakahara, T., Kamiyama, S. & Yamada, K. Kinetic studies on microbial activities in concentrated solutions. I. Effect of excess sugars on oxygen uptake rate of a cell-free respiratory system. *Agric. Biol. Chem.* **30**, 42–48, https://doi.org/10.1080/ 00021369.1966.10858549 (1966).
6. American Public Health Association (APHA), American Water Works Association (AWWA), Water Environment Federation (WEF). *Standard methods for the examination of water and wastewater*, (APHA, 2017).
